# The Anatomy of a Malpractice Lawsuit

**DOI:** 10.1093/asjof/ojad008

**Published:** 2023-02-01

**Authors:** Pradeep K Attaluri, Peter J Wirth, Steven P Moura, Ellen C Shaffrey, Venkat K Rao

**Affiliations:** From the Division of Plastic and Reconstructive Surgery, University of Wisconsin School of Medicine and Public Health, Madison, WI, USA; From the Division of Plastic and Reconstructive Surgery, University of Wisconsin School of Medicine and Public Health, Madison, WI, USA; From the Division of Plastic and Reconstructive Surgery, University of Wisconsin School of Medicine and Public Health, Madison, WI, USA; From the Division of Plastic and Reconstructive Surgery, University of Wisconsin School of Medicine and Public Health, Madison, WI, USA; From the Division of Plastic and Reconstructive Surgery, University of Wisconsin School of Medicine and Public Health, Madison, WI, USA

## Abstract

Medical malpractice lawsuits can be a source of emotional, physical, and financial distress for both providers and patients. A thorough understanding of the medical malpractice process's history and current landscape will help providers navigate malpractice challenges. Given the impact and prevalence of medical malpractice, in this paper, the authors sought to dissect the intricate anatomy of a medical malpractice lawsuit. This includes a comprehensive and detailed report of tort reform, the criteria of a medical malpractice suit, and a description of the court proceedings. In addition, the authors also performed an extensive review of the medicolegal literature and have provided recommendations for healthcare providers to avoid these lawsuits in their practice.

**Level of Evidence: 5:**

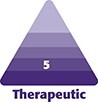

Medical malpractice cases continue to undermine physician practice patterns and increase insurance costs.^1,2^ It is estimated that medical malpractice expenditures in the United States amount to 56 billion dollars in healthcare spending on an annual basis.^1^ Medical malpractice lawsuits are also costly on an individual level, secondary to direct costs incurred by the surgeon or hospital and indirect costs related to lost time in clinical practice. While there have been vigorous attempts to curtail the number of frivolous lawsuits brought against healthcare providers, malpractice litigation continues to be a significant contributor to healthcare costs. It has substantial implications for healthcare providers named in a lawsuit, including the potential loss of licensure.^1^ In particular, surgeons face higher rates of malpractice claims when compared with nonsurgical providers.^1^ Yet, the lack of detailed understanding of the legal process in healthcare malpractice lawsuits puts providers in a vulnerable position.

Additionally, the delicate nature of medical and surgical patient care can create emotionally challenging situations when an unexpected outcome occurs. An unexpected outcome followed by a malpractice lawsuit can be incredibly distressing to physicians and lead to decreased personal and professional satisfaction.^3^ For these reasons, it is essential to identify the variables of malpractice lawsuits to amend our practice patterns to ensure patient and surgeon satisfaction.

Given the significant emotional and financial tolls on physicians, we sought to dissect the intricate anatomy of a medical malpractice lawsuit within the United States. A successful medical malpractice lawsuit must substantiate that a patient-physician relationship has been established, demonstrate a proximate cause, and show a breach in the standard of care. Unfortunately, most surgeons will likely encounter medical malpractice at some point in their careers, so having a general understanding of the malpractice process is beneficial. In this paper, we will identify the criteria of a successful medical malpractice suit, describe the court proceedings, highlight the importance of tort reform, and finally identify strategies to mitigate the risk of being named in a lawsuit (Figure 1).

**Figure 1.**
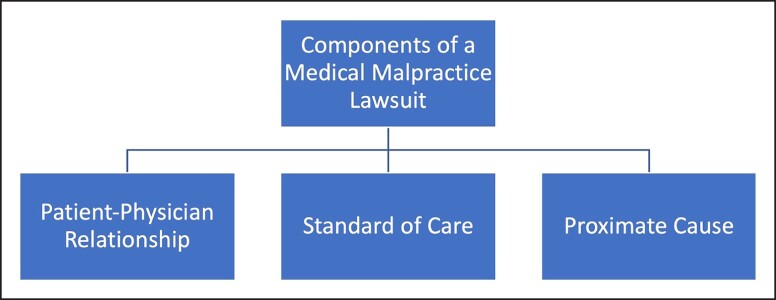
Three criteria needed to meet medical malpractice legitimacy.

## CRITERIA OF MEDICAL MALPRACTICE

### The Patient-Physician Relationship

The patient-physician relationship involves implicit rights, expectations, and limitations. To establish this relationship, the patient and physician must mutually consent to enter the relationship (except in certain circumstances, such as emergency care) in which a physician provides diagnostic and therapeutic services.^4^ Importantly, this relationship can be entered explicitly or implicitly. Explicit examples include patients receiving specific care from a physician in a professional setting, such as an outpatient consultation or an inpatient consulting service request. Implicit examples refer to informal discussions—including those through telephone or in social settings—which may be sufficient to establish the relationship. Explicit and implicit relationships can hold the physician accountable for adverse events, even with no direct patient care provided.^5,6^

The power of the patient-physician relationship was demonstrated in the case of *Adams v. Via Christi Regional Medical Center*, where a physician was found liable for the wrongful death of a former patient who died of a ruptured ectopic pregnancy. The physician offered medical advice for the patient's abdominal pain in early pregnancy during a phone call with the patient's mother. Despite testifying that he had not directly spoken to or examined the patient and was not an obstetric provider, he was ultimately found guilty on the basis that accepting the phone call and offering a medical opinion established this patient-physician relationship.^5,7^

This landmark case highlights the importance of understanding that physicians may decline to enter a patient-physician relationship when prospective patients seek care. This may be acceptable when the care falls outside the scope of practice, is not indicated, requires resources that are not readily available (provided the lack of resources are not discriminatory against specific patients), or the patient is abusive or threatening.^7^

An example of declining to enter a patient-physician relationship due to the scope of practice is highlighted in the *Estate of Kundert v. Illinois Valley Community Hospital* case. The mother of a newborn called the hospital for advice regarding high fevers in her child. She was connected with a healthcare provider who advised the mother that Tylenol (Johnson & Johnson, New Brunswick, NJ) and tepid baths should be used, but immediate medical attention was unnecessary. However, this provider also shared that the hospital did not have “the equipment or medical personnel to provide medical services to infants.” The child later died of bacterial meningitis. The court dismissed the case, arguing that informal opinions or advice do not equate to the acceptance of a patient. Furthermore, the statement regarding the lack of appropriate resources to care for the infant was a refusal to initiate a patient-physician relationship.^7^

These cases highlight the sometimes-ill-defined limits of the patient-physician relationship. Every plastic surgeon must consider instances in which we provide formal and informal medical advice, provide clear limits on what surgical procedures we can and cannot offer, and above all else, ensure that we provide appropriate care for our patients.

### Standard of Care

In addition to entering a patient-physician relationship, medical malpractice lawsuits hinge on the healthcare provider failing to meet the standard of care. While the definition of “standard of care” can vary from state to state, the general concept refers to a healthcare professional who can provide the same care as their peers in similar situations.^8,9^ Two landmark cases established the concept of standard of care: the cases of *T.J. Hooper* and *Helling v. Carey*.

While the case of Hooper was not directly related to medicine, its findings established the framework for evaluating the standard of care in relation to medical malpractice. In 1932, two barges sunk while Hooper was towing them with his tugboat during a storm. The owners of the barges sued Hooper on the grounds that he was unsafe given his tugboat lacked the proper equipment to review storm warnings. The justice ruled in favor of the barge owners, stating that if a practice or precaution is reasonable but not universally adopted, it can still be considered the standard of care.

In the case of *Helling v. Carey*, Helling sued her ophthalmologist Dr Carey for the loss of her eyesight secondary to glaucoma. While Carey won the trial and appeal, a final appeal in the Washington State Supreme Court overturned the verdict. The defense argued that the rate of glaucoma in patients younger than 40 years was exceedingly rare, and thus it was not standard to test patients with a tonometer. Since a tonometer is inexpensive and harmless, the Supreme Court determined it should have been used. As a result, many state legislatures began to legally define a standard of care.^10^

Establishing a breach in the standard of care throughout a medical malpractice lawsuit is achieved through various paths. The concept of “*res ipsa loquitor*” (ie, the thing speaks for itself) is one path in which the need for legal proceedings is obviated. The classic example in medicine is the retained sponge during surgery, which presents an obvious error. The need for an expert witness becomes apparent in cases where the error is not so obvious, which is a second way to establish a breach in the standard of care. The expert witness's job is translating complex medical concepts into layperson vernacular. The American Society of Plastic Surgeons Code of Ethics declares that its members must testify as expert witnesses when appropriate.^10,11^ In the United States, inherent biases can be introduced into the court as the plaintiff and defendant teams hire and pay their own expert witnesses. Though there have been recommendations to hire the same expert witness for both the plaintiff and defendant, this has not been adopted by the American judicial system.^11^

### Establishing Proximate Cause

Proximate cause is the determination that an event is related to an injury. The plaintiff's goal is to establish a proximate cause and make a case for compensation commensurate with their injuries. For causation to be established, the plaintiff or claimant must determine that there is: (1) the existence of duty by the physician, (2) a breach of that duty, (3) a resulting injury to the patient, and (4) a causal connection between the breach in duty and the injury.^12,13^

In the US legal system, 2 principles are used to establish the criteria above for causation: cause-in-fact and foreseeability. Cause-in-fact (also known as *sine qua non*) is commonly tested with the “but for” test. This is used to establish that event B would not have happened without event A. In the case of healthcare, this takes the form of “a substantial factor bringing about injury, without which the harm would not have occurred.”^14,15^ Cause-in-fact may be established in surgical cases—“without a rhinoplasty, the patient would not have a saddle nose deformity.” Using the same logic, “without a breast reduction, the patient would not have had malignant hyperthermia” would also establish cause-in-fact. However, this second example highlights a rare complication that surgeons or anesthesiologists cannot always predict; this highlights the second criterion, foreseeability.

Foreseeability suggests “that the act or omission complained of must be such that a physician using ordinary care would have foreseen that the event, or some similar event, might reasonably result therefrom.”^16^ As a result, cases that establish cause-in-fact also need to demonstrate that the result “could be reasonably anticipated.”^17^ In the example of malignant hyperthermia, the physician may not be able to anticipate this adverse reaction (ie, the patient has not had previous surgery and no family history of malignant hyperthermia). During malpractice litigation, plaintiffs will attempt to establish each of the aforementioned components of proximate cause.

## STRUCTURE OF THE COURT SYSTEM

The structure and hierarchy of courts are relatively constant across states; there are trial courts where civil disputes are tried, followed by appeals courts, with ultimate judicial power lying within the state's supreme court.^12^ Medical malpractice lawsuits are generally filed within the state trial court system. Unless there is a settlement before trial, each defendant has a right to a jury trial in the United States.^12,18^ This means that a group of impartial peers from the general public will be asked to assess the plaintiff's and defendant's arguments and decide based on legal precedent.^12^

Though there are cases that are heard by a jury, the majority of medical malpractice suits are settled outside of court. There are multiple reasons that medical malpractice lawsuits are settled outside of court, the most common being that the resource-intensive nature of trial is detrimental to clinical productivity.^19,20^ If it is determined that the cost of pursuing a trial is more costly than settling, the physician may be advised to settle the case.^15^ In the event that legal representation is needed to defend the physician, there are different options that are presented to the physician. Typically, an insurance carrier will retain counsel when a lawsuit is filed but will often honor a physician's request for a specific attorney. If the physician chooses to pursue a personal lawyer, the physician will incur the costs of those legal fees.^21^ There are instances in which an insurance carrier might not be willing to seek counsel for the physician due to conflict of interest. These situations include claims for punitive damages (insurance carriers are prohibited by law from paying punitive damages), the Bad Faith Doctrine in which the physician wants to settle but the carrier refuses, and uncovered claims such as when a physician is sued as both the attending physician on record and the medical director of a facility. Professional liability insurance only covers claims to represent the physician as a healthcare provider and not as a facility director.^21,22^

## OVERVIEW OF THE TRIAL

After a medical malpractice suit is filed, there is a laborious period of discovery, depositions, and interrogations, which includes the sharing of information between the plaintiff's and defendant's teams. The goal of this discovery period is geared toward achieving out-of-court agreements between the parties.^20^ If an out-of-court settlement is not achieved, the process of interrogations, depositions, and requests for documents will continue. The documents requested usually include the entire medical record, billing records, and other records related to the patient encounter.^12^ Once the documents are reviewed, an interrogatory (ie, line of questioning) is developed to gather information about the other party. This is followed by a deposition, in which a party is questioned under oath. By requiring parties to answer depositions under oath, the judicial system hopes to gather reliable information that can still promote an out-of-court settlement. Any information obtained during these legal proceedings can later be used in court. It is important to note that personal communications and any information in the medical chart can also be deemed discoverable. Information that is not discoverable includes that which is protected by peer review in an institution and information that is protected by attorney-client privilege.^23^ The protected peer review process includes conclusions reached by a hospital-wide committee after a thorough review of cases with established morbidity or mortality.^23^

If the case proceeds to trial, the plaintiff's legal team must convince the jury with a burden of proof that the physician was not providing adequate care. On the contrary, the defense will serve to negate the proof that the plaintiff presents. In medical malpractice lawsuits, there is a need only for “preponderance of evidence,” which translates to finding a greater than 50% probability that professional negligence occurred to find the physician guilty.^13,23^ After the court has determined the final damages, the losing party can file for an appeal. The appeal can be for a variety of reasons, including outcome of the trial or settlement of the trial. If the court mandates a monetary transaction, it must be reported to the National Practitioner Data Bank (NPDB). It is important to note that the pretrial exchange of monetary awards must also be reported to the NPDB.

## SIGNIFICANCE OF THE NATIONAL PRACTITIONER DATA BANK

The NPDB was established by Congress in 1986 to “improve the quality of health care by encouraging state licensing boards, hospitals, and other health care entities, and professional societies to identify and discipline those who engage in unprofessional behavior; and to restrict the ability of incompetent physicians, dentists, and other health care practitioners to move from state to state without disclosure of previous medical malpractice payment and adverse action history.”^24^ The NPDB consists of a system of reporting and querying by qualified entities, including medical malpractice payers, hospitals, state licensing boards, professional societies with formal peer review, and the US Drug Enforcement Agency.^24^ Medical malpractice payments are mandated to be reported when all 4 of the following criteria are met: payments are made by an organization, not an individual; on behalf of an individual, not a group; in settlement of a written complaint related to the lack of provision of care; and the request of money. All 4 of the aforementioned criteria must be met, irrespective of the amount of money or merit of the claim.^25^ This reporting to the NPDB is significant as hospitals can query this data bank as physicians apply for new jobs or to expand existing privileges.^24^

**Figure 2.**
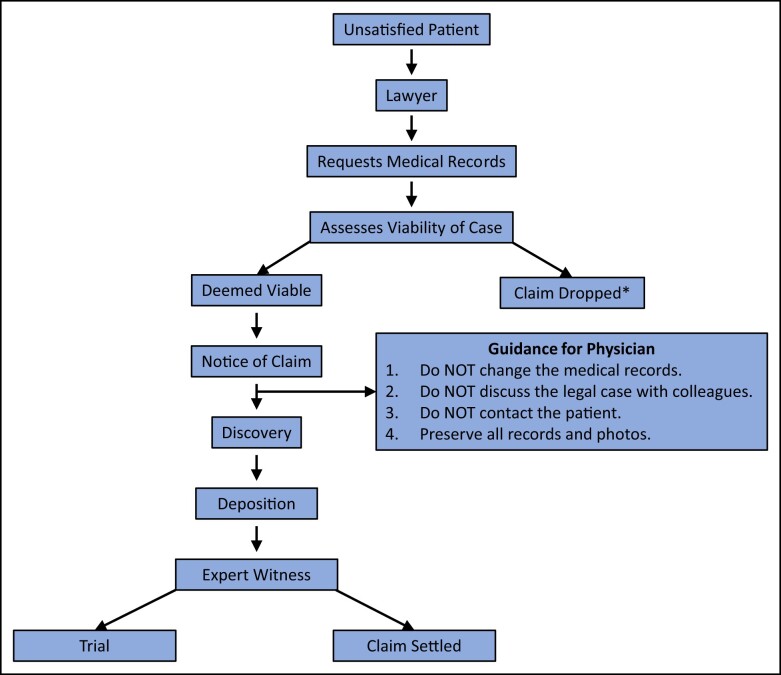
The summary of a medical malpractice lawsuit.

The NPDB has intentions to create a safe reporting and practicing environment in medicine, but there are imperfections within the system. The first argument is that payments made on behalf of physicians do not translate to malpractice. The legal process can prove to be expensive, therefore incentivizing insurance companies to settle with a payment rather than engaging in a costly malpractice trial.^24^ Secondly, the concept of a “corporate shield” has proven to be controversial.^24,25^ This concept occurs when payments are made on behalf of an institution rather than an individual to effectively bypass any entities with mandatory reporting responsibilities. This can prove unfair to practitioners in private practice, which is common in plastic surgery (Figure 2).^25^

## UNDERSTANDING TORT REFORM

Tort reform is one of the most instrumental strategies implemented to limit the number of medical malpractice lawsuits. Tort reform is described as a comprehensive list of legal strategies and provisions implemented by state legislatures to limit medical malpractice costs and ensure that patients injured by medical negligence can be fairly compensated (Table 1). Since the 1970s, tort reform has helped reduce the number of baseless medical malpractice lawsuits and limit excessive awards from the jury.^16,19^ These provisions include caps on noneconomic damage, limitations on the proportion of contingency fees collected by attorneys, and limitation on the collateral source rule.^20^

**Table 1.** Summary of Tort Reform Propositions

Reform measure	Description	Proposed effects
Caps on noneconomic damages	Limits amount of awards for noneconomic damages	Reduces indemnity payments Reduces defensive practices Limits insurance premiums
Contingency fee caps	Limits amount that the plaintiff's attorney can charge	Allow access to legal quality representation Decreases burden of malpractice cases on judicial system
Limiting collateral source rule	Allows deduction of award if injured patient received compensation from another source	Eliminates “double-dipping” by the plaintiff

### Caps on Noneconomic Damages

Caps on noneconomic damages limit the amount of monetary damages the plaintiff may collect for pain and suffering associated with the injury. These noneconomic damages attempt to capture the intangible losses suffered by the victim, separate from the financial losses that are compensable under the laws of various states.^20^ This cap has been set at $250,000US, an amount first established by the state of California under the Medical Injury Compensation Reform Act (MICRA).^25^ California was swift to create this cap after noticing an exodus of physicians from the state due to increasing insurance premiums resulting from high malpractice awards. This left a noticeable gap in healthcare throughout the state, forcing the legislature to create a more hospitable environment to practice medicine.^20^

Interestingly, California recently passed a law, AB-35, that will result in significant changes to the state's preexisting cap on noneconomic damages, including increases in the cap awarded to plaintiffs. Starting in 2023, cases not involving death will have a limit of $350,000US in 2023, with a gradual increase over the next 10 years to $750,000US by 2033.^25^ Those cases involving a patient's death will have an increased cap of $500,000US in 2023, with incremental increases over the next 10 years to reach $1,000,000US by 2033.

### Contingency Fee Caps

An instrumental component of tort reform was the creation of a contingency fee. A contingency fee is a form of payment to a lawyer for legal services but differs from a fixed payment, as lawyers only receive compensation when their client wins or settles the case.^26^ Some states further regulate the payment of lawyers in the form of a sliding scale that allocates higher contingency fees for the first $50,000US of the recovery and a reduction of the contingency fee for amounts exceeding $50,000US.^26^ Contingency fees are beneficial from a patient advocacy standpoint, as they allow access to quality legal representation motivated to work diligently for the plaintiff. Contingency fees have prompted lawyers to be judicious about case selection, which may limit the number of malpractice lawsuits. This fee system incentivizes lawyers to prefer defending plaintiffs when they believe a factual error has occurred, thereby decreasing the burden of malpractice cases on the judicial system.^12^ On the contrary, opponents believe that contingency fees deter the most able defense attorneys from taking on cases in which they believe the compensation is not worth the time and resources.^26^

### Limiting Collateral Source Rule

The collateral source rule, defined as a law that prevents the reduction of damages awarded to a plaintiff, has also been limited to avoid excessive awards given to the plaintiff.^27^ Common law states that the plaintiff is entitled to be fully compensated by the negligent party, even if the losses have already been paid by a separate party (ie, plaintiff's insurance company). The limitation of this collateral source rule decreases payouts by not allowing for the plaintiff to profit from losses that have already been appropriately compensated for by a separate party (ie, “double-dipping” from the plaintiff's insurance company). Most states have modified or abrogated the rule to be more compliant with the economic realities of today's healthcare system.^14,27^ In addition to the limitation of the collateral source rule, punitive damage has also been eliminated. Traditionally, in cases where the defendant has committed either intentional or negligent crimes against the plaintiff, the use of punitive damage was invoked to punish the defendant and deter future crimes of similar nature.

## COMMON MISTAKES IN PLASTIC SURGERY

A common adverse event in lawsuits against plastic surgeons is unhappiness with the result.^28^ While unhappiness may stem from subjective and objective measures of poor performance, it indicates a failure to meet a patient's expectations. The traditional informed consent process by which surgeons explain risks, benefits, alternatives, and expected preoperative and postoperative care does not necessarily address the concept of “happiness.” An effective physician-patient relationship is essential in decision making for the informed consent process. It has been advocated that an optimized physician-patient relationship leads to a better therapeutic strategy.^29^ Engaging the patient and understanding the emotional motivation behind a patient pursuing surgery leads to greater satisfaction in the informed consent process.^29^ In general, consent should be obtained either by the surgeon providing the treatment or by an associated clinician with sufficient knowledge of the surgery, potential complications, and alternative treatment options. The patient must also be competent, adequately informed, and not coerced. The minimum required informed consent process defined by the AMA consists of diagnosis, nature, and purpose of the recommended intervention, the burdens, risks, and expected benefits of all options, including forgoing treatment. This conversation must be meticulously documented in its totality to serve as verification of the informed consent process. Communication with the patient must be tailored to the patient's need that is material to the decision-making process, which places the onus on the physician to determine what is necessary based on medical judgment.^30^

Aesthetic surgeons have long recognized the importance of shared decision making (SDM) in developing a treatment plan that meets the patient's specific goals.^28^ As outlined by Elwyn et al, SDM includes 3 phases of “talk,” including (1) “Choice talk,” in which the patient is made aware that there are options available for treatment; (2) “Option talk,” in which the specific options for treatment of a condition are reviewed; and (3) “Decision talk,” in which patient preferences regarding treatment options are conveyed to reach a mutual decision.^31^ Yet, SDM was perceived to take too long during consultations, and many believe that they are already incorporating SDM in their practice.^20,30^ In a survey of ASAPS members, 63% of respondents believed that consent documents did not help teach patients the essential information they needed from the informed consent process.^20,32^ This highlights the difference between what plastic surgeons understand to be needed in a complete informed consent process, which includes SDM, and the current model, which is focused on the disclosure of information without demonstration of understanding. Inevitably, gaps in expectations and goals between the patient and surgeon exist, eroding the foundational relationship between patient and physician, and opening the door for future litigation.

## STRATEGIES TO AVOID A LAWSUIT

Most plastic surgeons will be named in a lawsuit at least once during their career. Yet, when compared to all physicians, plastic surgeons have some of the lowest indemnity payments.^18^ In a recent study by Boyd et al, plastic surgeons comprised only 3.31% of reported claims and 3.16% of paid claims between 2006 and 2016, and 58% of payments were less than $100,000US.^18^ These data highlight the relative infrequency with which claims against plastic surgeons are made, and payouts are low compared to other surgical specialties. The most prevalent claim against plastic surgeons was for failure to perform a procedure properly (60%), followed by failure to supervise, failure to recognize a complication, error in diagnosis, and treatment delay.^18^ While lawsuits may seem unavoidable, there are multiple strategies that plastic surgeons can utilize to ensure both patient safety and minimize the impact of a lawsuit and subsequent litigation (Table 2).

**Table 2.** Strategies to Optimize Patient Safety and Reduce the Risk of Litigation

Strategies
1. Maintain board certification by the American Board of Plastic Surgeons.
2. Ensure maintenance of mandatory certifications as determined by local, state, and federal laws for surgeons, nurses, and other clinic staff (ACLS, BLS, etc.).
3. Ensure surgical procedures are performed in accredited facilities when appropriate based on the procedure type, patient's medical comorbidities, and availability of emergency services or equipment.
4. Provide clear written information on surgical procedures that remains up to date with current practices.
5. Ensure informed consent is obtained with a focus on shared decision making and precise documentation of preoperative discussions.
6. Do not offer surgical procedures outside the scope of practice, are not clinically indicated, or in cases where risks outweigh benefits.
7. Do not offer surgical procedures if the surgeon or staff do not like the patient, the surgeon cannot understand the patient's goals, or the patient has unrealistic expectations.
8. Abide by Universal Protocol as outlined by the Joint Commission.

ACLS, advanced cardiovascular life support; BLS, basic life support.

## CONCLUSIONS

It is of paramount importance for plastic surgeons to have a thorough understanding of the medical malpractice landscape. Though most legal outcomes favor the surgeon, it is necessary to be cognizant of the potential for litigation in specific patient care situations. By implementing recommendations from our governing bodies, there is hope to reduce medical malpractice lawsuits and poor outcomes for the surgeon and patient. In an arena where change is the only constant, it is our field’s responsibility to educate our surgical trainees and providers to ensure optimal patient safety and the prevention of medical malpractice lawsuits.
